# Localized Perivascular Therapeutic Approaches to Inhibit Venous Neointimal Hyperplasia in Arteriovenous Fistula Access for Hemodialysis Use

**DOI:** 10.3390/biom12101367

**Published:** 2022-09-24

**Authors:** Allan John R. Barcena, Joy Vanessa D. Perez, Olivia Liu, Amy Mu, Francisco M. Heralde, Steven Y. Huang, Marites P. Melancon

**Affiliations:** 1 Department of Interventional Radiology, The University of Texas MD Anderson Cancer Center, Houston, TX 77030, USA; 2 College of Medicine, University of the Philippines Manila, Manila 1000, Philippines; 3 Grossman School of Medicine, New York University, New York, NY 10016, USA; 4 The University of Texas Southwestern Medical School, Dallas, TX 75390, USA; 5 The University of Texas MD Anderson Cancer Center UTHealth Graduate School of Biomedical Sciences, Houston, TX 77030, USA

**Keywords:** arteriovenous fistula, cell- and tissue-based therapy, chronic kidney disease, drug delivery systems, end-stage renal disease, hemodialysis, neointimal hyperplasia

## Abstract

An arteriovenous fistula (AVF) is the preferred vascular access for chronic hemodialysis, but high failure rates restrict its use. Optimizing patients’ perioperative status and the surgical technique, among other methods for preventing primary AVF failure, continue to fall short in lowering failure rates in clinical practice. One of the predominant causes of AVF failure is neointimal hyperplasia (NIH), a process that results from the synergistic effects of inflammation, hypoxia, and hemodynamic shear stress on vascular tissue. Although several systemic therapies have aimed at suppressing NIH, none has shown a clear benefit towards this goal. Localized therapeutic approaches may improve rates of AVF maturation by providing direct structural and functional support to the maturating fistula, as well as by delivering higher doses of pharmacologic agents while avoiding the adverse effects associated with systemic administration of therapeutic agents. Novel materials—such as polymeric scaffolds and nanoparticles—have enabled the development of different perivascular therapies, such as supportive mechanical devices, targeted drug delivery, and cell-based therapeutics. In this review, we summarize various perivascular therapeutic approaches, available data on their effectiveness, and the outlook for localized therapies targeting NIH in the setting of AVF for hemodialysis use. Highlights: Most systemic therapies do not improve AVF patency outcomes; therefore, localized therapeutic approaches may be beneficial. Locally delivered drugs and medical devices may improve AVF patency outcomes by providing biological and mechanical support. Cell-based therapies have shown promise in suppressing NIH by delivering a more extensive array of bioactive substances in response to the biochemical changes in the AVF microenvironment.

## 1. Arteriovenous Fistula Access for Hemodialysis Use

End-stage renal disease, which is defined as a stage of chronic kidney disease requiring kidney transplantation or renal replacement therapy, affects nearly 4 million individuals globally. Dialysis is still the predominant renal replacement therapy in most countries, and hemodialysis remains the most utilized dialysis modality [1]. For patients who require hemodialysis, vascular access is crucial to the efficiency and success of the procedure [2]. Types of vascular access include central venous catheters (CVCs), arteriovenous grafts (AVGs), and arteriovenous fistulas (AVFs). CVCs are primarily intended for short-term vascular access [3]. Compared with AVGs and AVFs, CVCs are associated with higher rates of complications, such as iatrogenic vascular injury, thrombosis, hematoma, and venous stenosis [4]. Complications from CVCs may then limit the number of venous access sites that can be utilized for the creation of AVGs and AVFs. Additionally, the risk of infection is heightened with CVCs; mortality related to infection is 41% higher in patients who receive dialysis through a CVC compared with patients who receive dialysis through an AVF [4]. Conversely, arteriovenous access methods such as AVGs and AVFs are more suited for long-term usage; however, AVGs require greater interventional requirements for maintenance and have a shorter functional lifespan than AVFs owing to increased risk of complications, such as infection and thrombosis [3,5,6]. Among the options for vascular access for hemodialysis, AVFs are associated with the highest patency rate, provided there is sufficient AVF maturation [7,8,9,10]. While endovascular and open surgical interventions are available to salvage AVFs that fail to mature and AVFs that suffer decreased flow rates over time, success rates for these salvage procedures are variable, their long-term durability is low, and the associated health care costs and burdens are necessarily increased [11]. Overall, approximately 20–60% of patients experience primary AVF failure, which underscores the need to explore strategies to improve AVF maturation and maintenance [12]. One such method is the suppression of venous neointimal hyperplasia (NIH), which interferes with AVF maturation.

In this review, we describe the pathophysiology of NIH and current clinical strategies to prevent AVF failure. We then summarize localized perivascular therapeutic approaches for supporting AVF maturation. Finally, we discuss future directions and further research needs in developing perivascular approaches that enhance AVF access outcomes.

## 2. Pathophysiology of Venous Neointimal Hyperplasia

The vein is immediately exposed to hemodynamic changes after AVF creation, which cause vascular remodeling and eventually maturation. Pathologic mediators disrupting the process of successful AVF maturation include turbulent wall shear stress (WSS), inflammation, and hypoxia, which may work synergistically to promote endothelial cell proliferation and inward migration of pro-inflammatory cells, smooth muscle cells, and myofibroblasts, eventually resulting in vascular stenosis [2].

### 2.1. AVF Creation and the Physiology of Maturation

**Figure 1:**
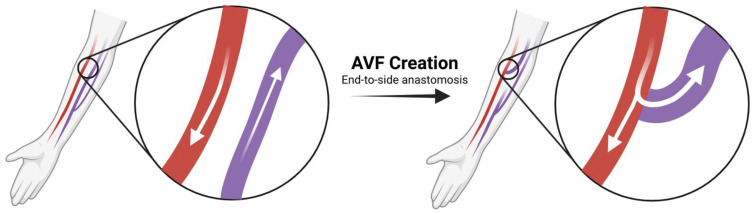
An end-to-side AVF anastomosis; immediately after surgery, the outflow vein (purple) experiences an increase in blood flow (Created with BioRender.com (accessed on 25 August 2022)).

During AVF creation, an artery is anastomosed to a vein through surgical intervention, leading to “arterialization” and dilatation of the venous outflow, which allows the fistula to be cannulated for hemodialysis (Figure 1). Following surgical creation, the AVF undergoes the process of maturation. Currently, however, no established consensus exists on the definition of a mature AVF [13]. The National Kidney Foundation-Kidney Dialysis Outcomes Quality Initiative defines a mature AVF as being at least 6 mm in diameter, less than 6 mm deep, and with a blood flow of at least 600 mL/min [14]. However, publications in the literature offer lower minimum flow rates of 300 mL/min to 400 mL/min for mature AVFs [15,16]. The lack of a consistent definition for AVF maturation limits accurate assessment of clinical data and may contribute to the wide range of AVF maturation success rates from different centers. Though definitions of AVF maturation vary, it is known that joining a high-pressure artery to a low-pressure vein for an AVF results in increased blood flow and blood pressure in the veins, which leads to an increase in WSS. Exposing the vein to this arterial environment necessarily requires physiologic vessel dilation and remodeling of the vein wall [16,17]. Indeed, a retrospective study by Hou et al. found venous distensibility to be predictive of AVF patency and usability in patients requiring hemodialysis, even more so than the baseline venous diameter, suggesting the importance of venous adaptability [18].

The increased WSS following AVF creation sets off a cascade of biochemical events. Endothelial nitric oxide synthase (eNOS) produces nitric oxide (NO), a potent vasorelaxant and vasodilator [17]. eNOS is upregulated in the venous limb following AVF creation, implicating NO in vascular adaptation to AVF creation [19]. Mouse AVF models have also suggested the importance of eNOS and NO in successful AVF maturation, with recent studies demonstrating that overexpression of eNOS results in favorable vascular profiles for AVF maturation, such as increased vein diameter and reduced NIH after AVF creation [20,21,22].

Another factor thought to be necessary to vascular remodeling is the fragmentation of the elastic lamina, likely caused by matrix metalloproteases (MMPs) through the degradation of proteins in the extracellular matrix (ECM) [2,17,23]. Lee et al. demonstrated that patients with successful AVF maturation had significantly higher levels of MMP-2 and MMP-9 than patients with unsuccessful AVF maturation [24]. However, the role of MMPs in AVF maturation remains elusive, with one study in a mouse AVF model demonstrating that MMP-9–knockout mice had an increased luminal area and reduced neointima formation in the AVF venous segment compared with wild-type mice [25]. Thus, while MMPs likely play a role in elastic lamina fragmentation, more evidence is needed to elucidate their exact roles in vascular remodeling and AVF maturation physiology.

Together, vascular dilation and remodeling are necessary to accommodate the increased blood flow and pressure resulting from AVF creation, enabling AVF cannulation for hemodialysis. Still, numerous other molecules are involved in vasodilatory and vascular remodeling responses and cascades, which may differ in arterial and venous limbs. Thus, increased research is needed to further elucidate the physiology of AVF maturation.

### 2.2. Pathways That Lead to Venous Neointimal Hyperplasia

**Figure 2:**
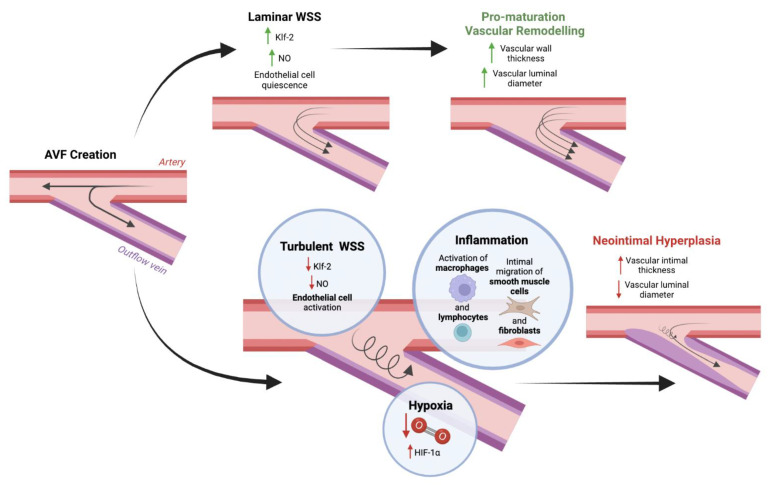
Pathways that lead to AVF maturation versus failure. Laminar WSS promotes outward remodeling. In contrast, the synergistic action of turbulent WSS, inflammation, and hypoxia promotes NIH. (Created with BioRender.com (accessed on 25 August 2022)).

One predominant cause of AVF failure is NIH, as this can cause venous stenosis and thrombosis, resulting in decreased blood flow through the AVF [26]. Indeed, multiple clinical and animal studies have reported failed AVF maturation resulting from venous stenosis caused by NIH [27,28,29,30,31]. Current evidence suggests that NIH after AVF creation occurs due to the synergistic action of turbulent WSS, inflammation, and hypoxia (Figure 2) [26,32].

In vitro and in vivo studies have demonstrated that endothelial cells exposed to laminar or unidirectional and high WSS maintain a quiescent phenotype through several mechanisms, which include expression of antimitogenic Kruppel-like factor 2 (Klf-2), stimulation of NO-mediated vasodilatory response, and stabilization of endothelial cells’ cytoskeletal architecture, alignment, and permeability [10,32]. The maintenance of the quiescent state is an essential component of AVF maturation, while uncontrolled endothelial activation that promotes inflammation contributes to NIH (Franzoni et al., 2016; T. Lee & Misra, 2016).

Inflammation arises from both local—surgical trauma and turbulent WSS—and systemic factors—surgical stress response and uremia—that leads to increased secretion of cytokines, which promote activation of pro-inflammatory macrophages and lymphocyte infiltration [2,32,33]. It also produces a stereotypical response that involves smooth muscle cell migration, proliferation, ECM deposition, and intimal expansion [34,35]. Studies have revealed various factors contributing to smooth muscle cells’ migration to the intima, including transcription factors nuclear factor erythroid 2-related factor 2 (Nrf2), growth factor platelet-derived growth factor (PDGF), cell cycle regulators p27 and p57, and microRNAs miR-221 and miR-222 [36,37]. Recent evidence suggests that smooth muscle cells play a pivotal role in balancing AVF maturation and failure. While differentiated smooth muscle cells contribute to medial wall thickening that aids maturation, dedifferentiated smooth muscle cells form a significant component of NIH [31,38]. Fibroblasts in the adventitial layer of the venous wall also mediate AVF maturation and failure. While fibroblasts secrete MMPs that contribute to pro-maturation vascular remodeling, they also contribute to NIH development by migrating from the adventitia to the inner wall and differentiating into myofibroblasts. Analysis of AVF failure in patients has demonstrated that these myofibroblasts form a significant portion of cells found in the neointima [31,39,40,41].

Emerging evidence also suggests that oxidative stress and the hypoxia pathway may contribute to NIH and venous stenosis development. Dissection injury during AVF creation induces oxidative stress and may induce the hypoxia pathway, as indicated by increased hypoxia-inducible factor 1-alpha (HIF-1α) expression, which is associated with venous stenosis [26,41,42]. Indeed, studies in rabbit AVF models have shown that administering supplemental oxygen to mitigate the hypoxic environment after AVF surgery corresponds with decreased HIF-1α expression and decreased NIH [43,44]. Together, these results suggest that the hypoxia pathway may also play a significant role in the development of NIH and venous stenosis, contributing to AVF failure.

## 3. Current Clinical Strategies to Prevent AVF Failure

Current strategies to prevent AVF failure in clinical practice include a thorough history and physical examination to identify preexisting vascular lesions that could compromise AVF maturation, adequate control of the patient’s comorbid illnesses, and optimizing surgical technique. The surgical technique covers the appropriate anastomotic configuration, apposition procedure, and suture method. Despite the implementation of these strategies, AVF failure rates remain high; even among patients who have a low-risk score based on formulas that are used to predict AVF failure and those who have undergone vascular mapping prior to AVF creation, high rates of failure are still observed [45]. There are also no recommended pharmacologic agents for preventing AVF failure. Although several investigational therapies have aimed at suppressing NIH, none has shown a clear benefit towards improving AVF patency outcomes. Systemic anticoagulation with heparin has increased bleeding complications with no benefit in AVF patency [46]. Several antiplatelet agents have also been studied for preventing AVF failure, but no agent has been shown to reliably decrease the failure rate. A large multicenter trial reported that aspirin use did not reduce AVF failure within 12 months of surgery [47]. Clopidogrel was shown to potentially reduce the frequency of AVF thrombosis yet did not affect AVF suitability for dialysis [48]. A recent Cochrane meta-analysis reported insufficient evidence to support the use of investigational agents, such as aspirin, clopidogrel, dipyridamole, dipyridamole plus aspirin, warfarin, sulfinpyrazone, glyceryl trinitrate (patch), and fish oil, for preventing arteriovenous access thrombosis [49]. It should be noted that the utilization of systemic agents for purposes of improving AVF maturation and patency is often limited by dose-related toxicity. Overall, evidence suggests that the current approach to AVF maturation and maintenance remains suboptimal. Hence, multiple studies have started focusing on developing localized therapies that could suppress the different pathways that lead to NIH and assist the maturation of the developing AVF while avoiding some of the pitfalls of systemic therapies.

## 4. Localized Perivascular Therapeutic Approaches

Localized perivascular therapeutic approaches have been developed to support the maturing fistula via a mechanical and/or biologic approach. Different perivascular therapy approaches (Table 1), including mechanical devices, targeted drug delivery, and cell-based therapeutics, have been made possible by the development of novel materials, including nitinol-based implants, polymeric matrices, polymeric scaffolds, hydrogels, and nanoparticles.

### 4.1. Mechanical Devices

**Figure 3:**
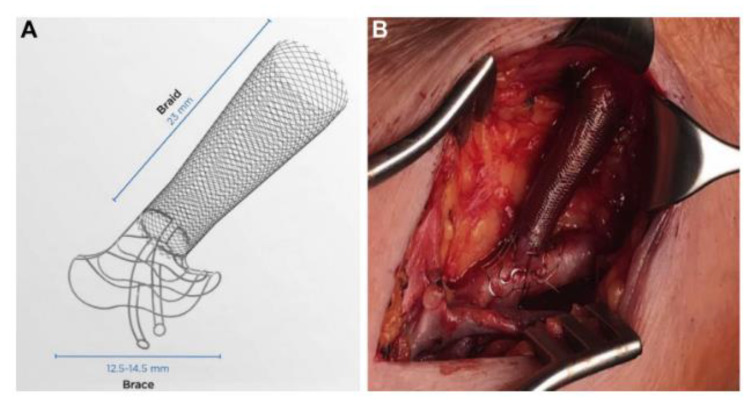
(**A**) The VasQ device is composed of a brace and a mesh braid. (**B**) An in vivo view of the device implanted over the anastomotic region of a brachiocephalic AVF. Images are reprinted, with permission, from Karydis et al. (2020) [50].

The use of supportive mechanical devices aims to minimize NIH by decreasing turbulence through the fistula and modulating pathologic vascular WSS [62]. The VasQ (Laminate Medical Technologies Ltd., Tel Aviv-Yafo, Israel), which consists of two components made of nitinol (a nickel and titanium alloy), is an example of an externally applied, perivascular device placed to support the anastomotic site externally; the first component is a brace that wraps the artery, while the second component is a mesh braid that encloses the juxta-anastomotic vein (Figure 3). A trial involving 40 patients with kidney failure referred for the creation of a brachiocephalic fistula reported significantly larger venous luminal diameters in the treatment group versus the control group at three months (8.27 ± 2.2 versus 6.69 ± 1.8 mm; *p* = 0.03) and six months (9.6 ± 2.5 versus 7.56 ± 2.7 mm; *p* = 0.03). In addition, the functional patency (defined as successful two-needle cannulation for at least two-thirds of all dialysis sessions) at six months after brachiocephalic fistula formation was significantly higher in the treatment group (n = 14 of 14 patients, 100%) than in the controls (n = 5 of 9 patients, 56%; *p* = 0.01) [50]. One limitation of the study is the reduced power for the endpoints due to the trial’s small sample size. Hence, more extensive studies are recommended to confirm this clinical benefit.

**Figure 4:**
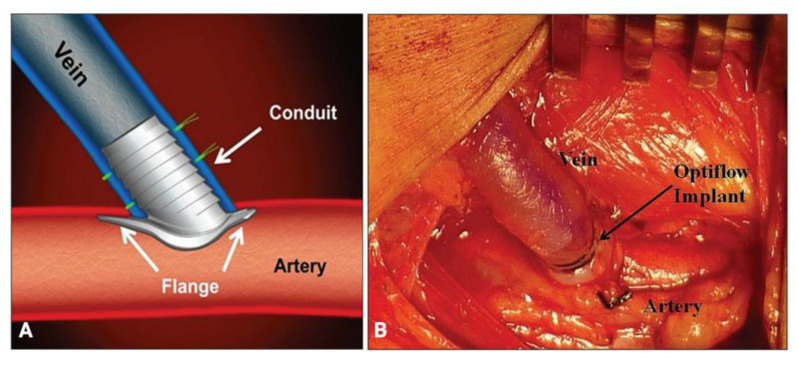
(**A**) The Optiflow device is composed of a flange, a conduit, and platinum bands. (**B**) An in vivo view of the device implanted within the anastomotic region of an AVF. Images are adapted, with permission, from Chemla et al. (2014) [63].

As opposed to the external VasQ device, Optiflow (Bioconnect Systems, Inc., Philadelphia, PA, USA), a single-piece implant made of siliconized polyurethane, is placed inside the anastomosis of the AVF to maintain a predetermined luminal diameter and improve blood flow. It consists of a flange, which anchors the device to the artery, and a conduit, which shunts the blood to the vein; it also has platinum bands that allow for fluoroscopic visualization (Figure 4). A study involving 41 patients with end-stage renal disease who underwent AVF creation using the Optiflow device reported unassisted patency rates of 93% (n = 38 of 41 patients), 88% (n = 36 of 41 patients), and 78% (n = 32 of 41 patients), at 14, 42, and 90 days, respectively, as well as mean venous diameters of 6.4 (±1.0), 7.2 (±1.5), and 7.6 (±1.8) mm at 14, 42, and 90 days, respectively [63]. No study has directly compared the effects on AVF maturation of an externally applied device versus an intravascular implant.

### 4.2. Targeted Drug Delivery

Localized exposure to pharmacologic agents is postulated to protect the anastomotic region from NIH via delivery of higher doses of NIH-suppressing agents while minimizing systemic side effects. Pharmacologic agents are delivered onto the adventitial layer via various mechanisms, including topical administration, drug-eluting polymeric scaffolds, or nanoparticle-based drug delivery. Examples of agents studied for perivascular administration include sirolimus, paclitaxel, simvastatin, 1α,25-dihydroxyvitamin D3, vonapanitase, β-aminopropionitrile, bevacizumab, and 4-amino-1,8- naphthalimide.

#### 4.2.1. Sirolimus

Sirolimus, also known as rapamycin, is an antiproliferative agent that belongs to a class of drugs known as mammalian/mechanistic target of rapamycin (mTOR) inhibitors. In clinical practice, sirolimus has been shown to inhibit NIH associated with stent placement for the management of coronary artery disease when administered locally to the arterial wall via an endovascular drug-eluting stent [64]. In a 2012 first-in-human study by Paulson et al., it was shown that the perivascular application of Coll-R, a sirolimus-eluting collagen membrane, during graft surgery could safely deliver sirolimus to the vessel wall without delivering an immunosuppressive dose to the systemic circulation [51]. Local delivery of sirolimus produced higher rates of 12- and 24-month primary unassisted patency (76 and 38%, respectively) than were reported in previous studies (23 to 43% at 12 months [65,66]) and a lower rate of thrombosis (0.37 events per patient-year) than previously reported (0.41 to 1.03 events per patient-year [67]. The efficacy of perivascular delivery of sirolimus in promoting AVF maturation is currently being evaluated. Sirogen (Vascular Therapies, Inc., Cresskill, NJ, USA), a novel collagen-based drug delivery system for sirolimus specifically designed for AVF, is undergoing phase 3 investigation following a previous phase 2 study that reported a maturation success rate of 87% [52].

#### 4.2.2. Paclitaxel

**Figure 5:**
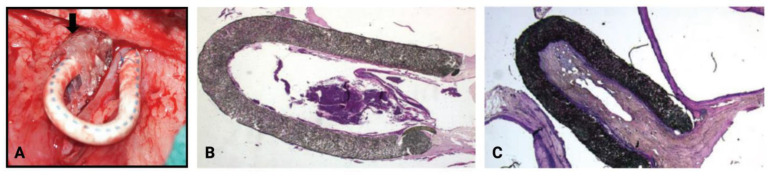
(**A**) Placement of perivascular paclitaxel wraps at the graft-vein anastomosis in a swine model. (**B**,**C**) Hematoxylin and eosin (H&E) staining (magnification, 100x) shows a marked decrease in NIH at the graft-vein anastomosis when treated with a paclitaxel-loaded perivascular wrap (**B**) as compared with a control wrap (**C**). Images are reprinted, with permission, from Kelly et al. (2006) [68].

Paclitaxel is an antiproliferative chemotherapeutic agent that interferes with cell replication via stabilization of microtubules and distortion of mitotic spindles; it is highly effective at blocking the proliferation of neoplastic cells, but its utility in non-neoplastic growth, such as NIH and vascular stenosis, is limited by its high toxicity following systemic administration [68]. Initial data obtained from local drug delivery in animal models of AVGs have been promising. In 2006, Kelly et al. developed a material consisting of a solvent-cast ethylene vinyl acetate (EVA) polymeric matrix containing 5% w/w paclitaxel and 15% w/w polyethylene glycol (PEG) 4000 [68]. The paclitaxel release profile of this matrix, or wrap, consisted of a burst release of 40% of the loaded paclitaxel within the first 72 h, followed by continuous release of 10% of the paclitaxel over the next 11 days, mirroring the pattern of cellular activation in polytetrafluoroethylene (PTFE) graft stenosis [68]. The placement of paclitaxel-loaded wrap around the graft-vein anastomosis in a swine model is shown in Figure 5. Paclitaxel was not detected in the systemic circulation of any pigs (n = 6), even during the burst-release phase (day 2), and no evidence of hematological toxicity was observed. Moreover, no luminal stenosis (<0.5%) was observed at any of the graft-vein anastomoses treated with paclitaxel-eluting wraps, whereas control wraps without paclitaxel showed mean luminal stenosis of 1.34 ± 1.07%, 20.59 ± 8.89%, and 49.46 ± 28.87% in the early (10 days), middle (24 days), and late (36 days) time point groups, respectively [68].

**Figure 6:**
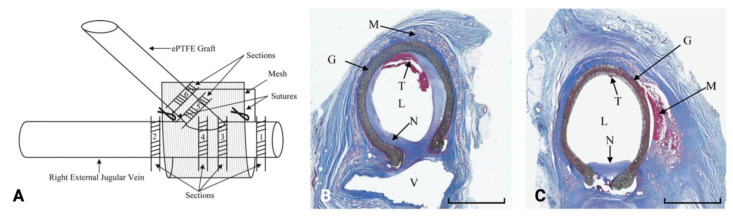
(**A**) Schematic diagram of the right external jugular vein and ePTFE graft anastomosis with applied mesh and histologic sections. (**B**,**C**) Photomicrographs of representative sections from the zero-dose mesh control group (**B**) and the 1.2-μg/mm2 paclitaxel mesh group (**C**). G, ePTFE graft; L, lumen; M, mesh; N, neointima; T, thrombus; V, vein. Bars represent 4 mm. Images are reprinted, with permission, from Kohler et al. (2007) [69].

Perivascular administration of paclitaxel was further investigated in 2007 by Kohler et al. using a polyglycolic-polylactic acid mesh (Angiotech Pharmaceuticals, Inc., Vancouver, BC, Canada) that fully degrades via hydrolysis in vivo in 60 to 90 days [69]. The paclitaxel elution profile of this mesh consisted of a burst release of paclitaxel during the first 24 h, followed by gradual release over the next 8 days. Three doses of paclitaxel were tested. At 1 week, 60% of the loaded paclitaxel was released from the meshes with the lower two doses (0.3 and 0.7 μg/mm^2^), while 45% of the paclitaxel was released from the highest-dose mesh (1.2 μg/mm^2^) [69]. The investigators then tested the efficacy of the paclitaxel-eluting mesh in a sheep model of NIH for 8 weeks; commercially available expanded PTFE (ePTFE) grafts were placed between the left common carotid artery and right jugular vein, and the paclitaxel-eluting wrap was placed around the graft-vein anastomosis (Figure 6A). The addition of mesh alone resulted in thicker fibroplasia encapsulating the graft and an increase in perigraft macrophages, which were concentrated around the disintegrating mesh debris. NIH was extensive in the group with no mesh (10.5 ± 6.8 mm^2^) and the group with zero-dose mesh (6.4 ± 3.2 mm^2^; Figure 6B), while the neointimal area was significantly lower in all paclitaxel mesh groups (ranging from 0.9 ± 1.4 to 1.3 ± 1.5 mm^2^; *p* < 0.008; sample, Figure 6C) [69]. Similarly, the mural thrombus area, amount of luminal stenosis, and capillary density decreased in the paclitaxel mesh groups [69]. The encouraging results in this study led to a phase 1/2 clinical trial headed by Angiotech Pharmaceuticals, Inc., in 2008; however, the study was terminated early because of a higher number of graft infections in the treatment arm than in the control arm [62]. The utility of paclitaxel-eluting wrap specifically for supporting AVF maturation has yet to be evaluated.

#### 4.2.3. Simvastatin

Simvastatin is a competitive inhibitor of 3-hydroxy-3-methylglutaryl-coenzyme A (HMG-CoA) reductase, the enzyme that catalyzes the rate-limiting step in the biosynthesis of cholesterol. It is a widely prescribed cholesterol-lowering agent, and it has been shown to reduce inflammation at the site of the coronary plaque, inhibit platelet aggregation, and inhibit the coagulation cascade [70]. Because of these properties, many studies have explored the effect of simvastatin in arteriovenous access maintenance. An initial investigation by Zhang et al. on a murine vein graft model demonstrated that simvastatin, administered subcutaneously 72 h before and then daily after surgery, significantly inhibited smooth muscle cell proliferation and vein graft NIH in a dose-dependent manner [71]. In 2013, a study by Janardahanan et al. on a murine AVF model reported that a series of intraperitoneal injections of simvastatin before AVF creation significantly reduced NIH and increased the vascular luminal diameter via modulation of smooth muscle cell activation, proliferation, and migration [39]. In 2016, Chang et al. found that statin use among patients with AVF reduced the risk of requiring AVF re-creation by 21% [72].

Although the systemic administration of statins has had promising results, patients may experience adverse effects, such as myopathy and hepatotoxicity, from high-dose therapy [53]. Localized perivascular delivery is posited to allow the delivery of high-dose treatment on the vascular wall without significantly increasing the risk for systemic toxicity. Zhao et al. recently implemented periadventitial delivery of simvastatin in a murine AVF model by using cyclodextrin microparticles and found that this strategy reduced the expression of known NIH mediators; the expression of vascular endothelial growth factor-A (VEGF-A), transforming growth factor-beta 1 (TGF-β1), and monocyte chemoattractant protein-1 (MCP-1) significantly decreased by 84% (*p* < 0.01), 69% (*p* < 0.05), and 79% (*p* < 0.05), respectively [53]. There were also significant decreases in the average neointimal area in the simvastatin-treated group (21,669 ± 8636 μm^2^) versus the control group (52,910 ± 21,738 μm^2^; *p* < 0.05) and in neointimal cell density in the simvastatin-treated group (15,578 ± 3966/mm^2^) versus the control group (21,622 ± 1911/mm^2^; *p* < 0.05) [53]. 

#### 4.2.4. 1α,25-Dihydroxyvitamin D3

1α,25-dihydroxyvitamin D3 is an inhibitor of immediate early response 3 (IER3) expression [73]. IER3 expression, upregulated by turbulent WSS on endothelial cells and mechanical stretching of smooth muscle cells in AVFs, has been shown to promote inflammation, fibrosis, and cellular proliferation and migration following AVF creation; conversely, IER3-knockout mice were demonstrated to have suppression of NIH [54]. Brahmbhatt et al. studied the periadventitial application of 1α,25-dihydroxyvitamin D3–coated nanoparticles composed of polylactic-co-glycolic acid (PLGA) and Pluronic F127 hydrogel in a murine AVF model, and they found that compared with the control group, this approach significantly reduced both IER3 gene expression (by 35%; *p* < 0.05) and the average ratio of neointima to media plus adventitia (by 34%; *p* < 0.05) [54]. Singh et al. further investigated perivascular nanoparticle-based administration of 1α,25-dihydroxyvitamin D3 in a porcine AVF model and also reported a significant reduction in IER3 expression at days 3 (86%; *p* < 0.004) and 28 (38%; *p* < 0.02) in the treatment group compared with the vehicle group; after 28 days, the treatment group, compared with the vehicle group, also showed a significantly higher average luminal area in the outflow vein (12.31 ± 1.93 mm^2^ versus 5.08 ± 0.54 mm^2^; *p* = 0.02), lower average neointimal area (0.41 ± 0.07 mm^2^ versus 1.65 ± 0.58 mm^2^; *p* = 0.01), and lower cell density in the neointima (4244.14 ± 922.45/cm^2^ versus 13,083 ± 2334.08/cm^2^; *p* = 0.03) [55].

#### 4.2.5. Vonapanitase

Vonapanitase, previously known as PRT-201, is a recombinant human chymotrypsin-like elastase family member 1 (CELA1) that cleaves the protein elastin [74]. It is hypothesized to contribute to AVF maturation by augmenting the elastin-fragmenting activity of endogenous elastases or MMPs, which is essential in outward vascular remodeling [56]. In PATENCY-1, a double-blind, placebo-controlled trial that treated 311 patients who underwent radiocephalic AVF creation, topical application of vonapanitase onto the anastomotic site for 10 min immediately after fistula creation did not improve primary patency, defined as the time from fistula creation to thrombosis or salvage procedure, but was associated with increased secondary patency and use for hemodialysis, defined as the ability of the fistula to be cannulated with two needles for at least 90 days [56]. The Kaplan–Meier estimates of the 12-month primary patency rates were 42% for the vonapanitase group and 31% for the control group (*p* = 0.25), and for 12-month secondary patency, the estimates were 74% for the vonapanitase group and 61% for the control group (*p* = 0.048). The proportions of patients with combined unassisted plus assisted use for hemodialysis were 64% for the vonapanitase group and 44% for the control group (*p* = 0.006) [56]. These findings were re-examined in the PATENCY-2 trial, which treated 603 patients using a similar protocol, but vonapanitase treatment failed to achieve statistically significant improvement in AVF outcomes [57].

#### 4.2.6. β-Aminopropionitrile

β-aminopropionitrile (BAPN) is an irreversible inhibitor of lysyl oxidase, an enzyme that catalyzes the deamination of lysine and hydroxylysine residues, an essential step in the formation of covalent cross-links in ECM proteins. Hernandez et al. found significantly higher lysyl oxidase deposition (5.3% versus 1.69% of vascular wall area; *p* = 0.03) and trivalent cross-linking density (0.061 µmol/mg versus 0.041 µmol/mg of dry tissue weight; *p* = 0.03) in veins of AVFs that failed versus those that successfully matured [58]. They hypothesized that BAPN administration could improve AVF patency and found that local delivery of BAPN in rat AVFs through an electrospun scaffold significantly decreased wall fibrosis (34.79 ± 3.83 versus 47.17 ± 8.65%; *p* = 0.03) and demonstrated a nonsignificant trend toward increased blood flow (34.03 ± 17.32 versus 24.80 ± 7.60 mL/min; *p* = 0.28) versus vehicle-loaded scaffolds at day 21 [58]. These findings indicate that local BAPN administration via perivascular scaffolds may improve AVF maturation, but more studies are needed to substantiate this hypothesis. 

#### 4.2.7. Bevacizumab

Inhibition of the NIH mediator VEGF-A is a potential strategy for promoting AVF maturation. Yang et al. studied the effect on AVF maturation of a lentivirus-delivered small-hairpin RNA that inhibits VEGF-A expression and found that this technique significantly increased the mean vessel luminal area of mouse AVFs [40]. Next, the investigators demonstrated that the peritoneal administration of bevacizumab, a humanized monoclonal antibody to VEGF-A, caused the same response, which further supported the efficacy of VEGF-A suppression on AVF maturation. The utility of locally delivered bevacizumab for AVF maturation has yet to be evaluated. Nonetheless, these findings highlight the potential role of both antibody-based and RNA-based therapies in AVF maturation [40].

#### 4.2.8. 4-Amino-1,8-naphthalimide

Natural vascular scaffolding (NVS) therapy is a novel technology that relies on the photoactivation of locally delivered 4-amino-1,8-naphthalimide to interlink collagen and elastin. In a recent study by Shiu et al., this approach significantly increased the open luminal area (4.2-fold; *p* = 0.014) and the percentage of the open luminal area (2-fold; *p* = 0.009) of the venous limb of rat AVFs versus the phosphate-buffered saline (PBS) control group [59]. They also found that NVS treatment changed the configuration of collagen fibers—producing a trend toward perpendicular alignment to the lumen circumference and more defined features—which suggests that this approach exerted changes in collagen arrangement that may influence AVF maturation [59].

### 4.3. Perivascular Cell-Based Therapeutics

The use of cells that have the ability to release a wide variety of substances that could potentially suppress NIH has been explored in recent studies. As opposed to localized perivascular drug delivery, cell-based therapies could deliver a more extensive array of bioactive substances in response to the biochemical changes in the AVF microenvironment. Examples of cells studied for perivascular administration include endothelial cells and mesenchymal stem/stromal cells.

#### 4.3.1. Endothelial Cells

Blood outgrowth endothelial cells (BOECs; a subgroup of circulating progenitor cells with an endothelial cell phenotype) have been reported to have therapeutic effects on models of vascular stenosis via modulation of hypoxia and vascular remodeling [75,76]. Based on these effects, Hughes et al. investigated the impact of periadventitial transplantation of BOECs on PTFE grafts in a porcine model of chronic renal insufficiency [77]. Autologous BOECs were transplanted to the adventitia of the AVG anastomosis using a polyglycolic acid (PGA) scaffold; after 14 days of exposure to BOECs, the vein-to-graft anastomosis showed a significant increase in MMP activation (*p* = 0.043) and a decrease in HIF-1α expression (*p* = 0.0475), and the mean intima-to-media ratio of the vessels transplanted with BOECs (5.5 ± 1.8) was around 50% less than that of the control vessels (11.4 ± 2.2, *p* < 0.05) [77].

**Figure 7:**
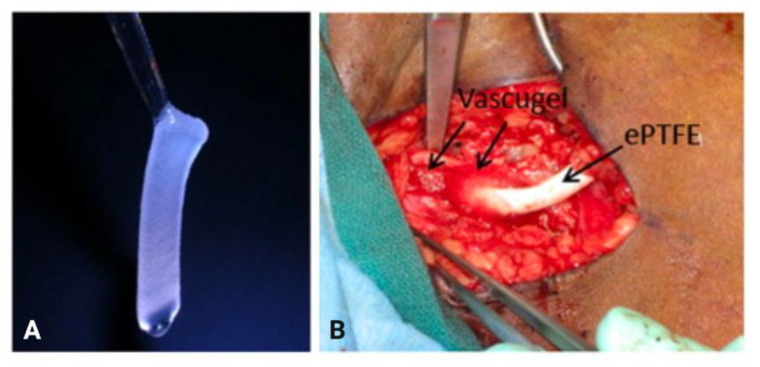
(**A**) Sample of a Vascugel sponge used in the trial. (**B**) Sponge placement at the anastomotic site of an AVG. Images are reprinted, with permission, from Conte et al. (2009) [60].

In 2009, Conte et al. reported the results of a multicenter phase 1/2 trial that evaluated the safety and efficacy of Vascugel (Pervasis Therapeutics, Inc., Cambridge, MA, USA), a novel cell-based product composed of allogeneic aortic endothelial cells in gelatin sponges, for arteriovenous access maturation [60]. In this trial, Vascugel sponges (1.0 × 4.0 × 0.3 cm) were applied to the anastomotic sites of AVGs and AVFs (Figure 7), with each sponge containing approximately 1.23 × 106 human aortic endothelial cells with at least 90% viability. After 24 weeks, there were no statistically significant differences in primary or assisted primary patency between the treatment and control matrix groups [60].

#### 4.3.2. Mesenchymal Stem/Stromal Cells

Mesenchymal stem cells (MSCs) are propagated from different sources, including bone marrow, adipose tissue, and cord blood [78], and have anti-inflammatory properties that promote homeostasis, repair, and regeneration in the setting of vascular injury [79]. MSCs have also been found to reduce fibrosis in the heart [80], lung [81], liver [82,83], and kidney [84] following cell transplantation in experimental animal models [85]. In addition, MSCs have been shown to home to areas of myocardial infarction to reduce hypoxic injury [86,87]. Because of these different properties, MSCs have generated interest as an emerging therapeutic modality in alleviating pathologic processes in vascular injury.

**Figure 8:**
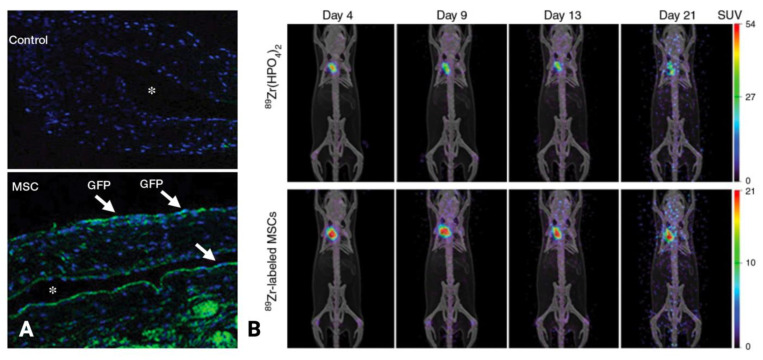
(**A**) Photomicrographs (original magnification, 20%#xD7;) show the localization of GFP-labeled human adipose tissue-derived MSCs (arrows) 7 days after injection into the adventitia of the outflow vein; ∗ = lumen. (**B**) Serial PET images of ^89^Zr distribution in mice after adventitial delivery of ^89^Zr-labeled MSCs or ^89^Zr (HPO_4_)_2_. The anatomic reference skeleton images are formed using the mouse atlas registration system algorithm with information obtained from the stationary top-view planar x-ray projector and a side-view optical camera. SUV = standardized uptake value. Images were adapted from Yang et al. (2015) [61].

Yang et al. hypothesized that adventitial transplantation of human adipose-derived MSCs to the outflow vein of the AVF at the time of creation would decrease pro-inflammatory cytokines and thereby inhibit NIH [61]. In their study, human MSCs were isolated from healthy donors and were transplanted as xenografts to immunodeficient B6.Cg-Foxn1nu/J mice by direct injection into the adventitia of the outflow vein at the time of AVF creation (carotid artery to jugular vein end-to-side anastomosis). MSCs were present in the adventitia on day 7 after administration; by day 21, they were no longer visualized using fluorescence imaging of tagged GFP but could still be visualized by PET imaging using 89Zr labeling (Figure 8). Compared with control vessels, the MSC-transplanted vessels demonstrated a 62% reduction in *MCP-1* expression (*p* = 0.029), 62% reduction in HIF-1α expression (*p* = 0.0005), 415% increase in mean luminal area (*p* = 0.011), 77% reduction in mean neointimal area (*p* = 0.013), and 83% reduction in neointimal cell density (*p* < 0.0001) [61].

## 5. Future Directions

A wave of innovation in localized therapeutics has been sparked by the unmet clinical need for modalities that can enhance AVF access outcomes. Different perivascular approaches are being developed, and more extensive studies pertaining to these approaches are expected in the coming years. Several pharmacologic agents delivered locally, such as paclitaxel and 1α,25-dihydroxyvitamin D3, have not been tested in humans yet, but a phase 3 trial of sirolimus embedded in a novel collagen-delivery system is currently being conducted (ClinicalTrials.gov ID: NCT05425056). A phase 1/2 trial is also assessing the effect of autologous adipose-derived MSCs delivered locally to the adventitia of newly created AVFs (NCT02808208). Although a trial that evaluated the impact of the VasQ device (Laminate Medical Technologies Ltd., Tel Aviv-Yafo, Israel) on AVF patency outcomes concluded recently, its statistical power was limited by its sample size, and a more extensive study should be conducted to validate the reported improvement in outcomes. Recent negative trials involving vonapanitase and Vascugel (Pervasis Therapeutics, Inc., Cambridge, MA, USA) for the purpose of improving AVF patency [57,60] highlight the need to improve our understanding of the use of these agents so that modifications or alternative strategies can be implemented.

Attempts to successfully deliver drugs locally at the arteriovenous anastomosis have been limited by the relatively rapid washout of the therapeutic agent. The development of novel materials, such as polymeric scaffolds and nanoparticles [88], is paving the way for the local delivery of novel pharmacologic agents and cell-based therapies, with the goal of prolonging drug and/or cellular contact with the AVF. Electrospinning, first reported in the early 1930s, has gained attention recently due to a surging interest in nanotechnology. This method can quickly fabricate various polymers into fibrous scaffolds with diameters at the submicron level [89]. While perivascular wraps loaded with antiproliferative agents, such as sirolimus and paclitaxel, have been used to reduce NIH [90], their utility in the delivery of cell-based therapy has yet to be explored. Nanofibrous electrospun polymers have been observed to recapitulate the scale and three-dimensional arrangement of collagen fibrils in the ECM [91,92]. Furthermore, their highly porous microstructures and extremely high ratio of surface area to volume have been shown to facilitate cellular infiltration, as well as nutrient and gas exchange, and to maximize cell-surface interactions [93]. Hence, the use of perivascular scaffolds in cell-based therapies may improve the retention, integration, and functional performance of transplanted cells. Using external reinforcement and self-sealing polymers, which have been used in the development of modified AVGs, perivascular scaffolds can also be enhanced to optimize their capacity to provide mechanical support. Despite the fact that bioresorbable polymers are naturally radiolucent, they can also be combined with nanoparticles to give them radiopacity, enabling the tracking of their deployment and breakdown over time [94]. An all-in-one electrospun perivascular wrap that delivers drugs and/or cells locally while also providing mechanical support for ideal hemodynamics should be possible with the integration of emerging technologies. Overall, the current challenges in developing perivascular therapies open further innovation opportunities. As our knowledge of the pathways that lead to NIH advances, the various approaches under investigation will be refined, and more innovative strategies will be devised.

## Figures and Tables

**Table 1:** Localized perivascular therapeutic approaches for supporting AVF maturation.

Approach	Specific Therapy	Development Stage	Results	Reference or ClinicalTrials.gov ID
Mechanical device	VasQ	Clinical (Phase 1/2)	Significantly increased venous luminal diameters at three and six months and increased functional patency at six months following brachiocephalic fistula formation	Karydis et al. (2020) [50]
Targeted drug delivery	Sirolimus	Clinical (Phase 1/2)	Local delivery using a collagen membrane produced 12- and 24-month primary unassisted patency rates of 76% and 38%, respectively, and a thrombosis rate of 0.37 events per patient-year	Paulson et al. (2012) [51]
		Clinical (Phase 2)	Local delivery using collagen-based technology demonstrated an AVF maturation success rate of 87%	Clair et al. (2019) [52]
		Clinical (Phase 3)	Ongoing trial	NCT05425056
	Simvastatin	Preclinical (Murine)	Periadventitial delivery using cyclodextrin microparticles significantly reduced the expression of VEGF-A, TGF-β1, and MCP-1 and reduced the average neointimal area and neointimal cell density	Zhao et al. (2020) [53]
	1α,25-dihydroxyvitamin D3	Preclinical (Murine)	Periadventitial delivery using PLGA and Pluronic F127 hydrogel significantly reduced *IER3* gene expression and the average ratio of neointima to media plus adventitia	Brahmbhatt et al. (2014) [54]
		Preclinical (Porcine)	Perivascular nanoparticle-based administration significantly reduced IER3 expression on days 3 and 28, increased the average luminal area in the outflow vein, and decreased the average neointimal area	Singh et al. (2021) [55]
	Vonapanitase	Clinical (Phase 1/2)	Topical application onto the anastomotic site did not improve primary patency but increased secondary patency and use for hemodialysis	Bleyer et al. (2019) [56]
		Clinical (Phase 1/2)	Topical application produced no significant improvement in AVF outcomes	Peden et al. (2022) [57]
	β-aminopropionitrile	Preclinical (Murine)	Local delivery through an electrospun scaffold significantly decreased wall fibrosis and demonstrated a nonsignificant trend toward increased blood flow on day 21	Hernandez et al. (2021) [58]
	4-amino-1,8-naphtalamide	Preclinical (Murine)	Local delivery and photoactivation significantly increased the open luminal area and % of the open luminal area of the venous limb of AVFs	Shiu et al. (2021) [59]
Cell-based therapeutics	Endothelial cell	Clinical (Phase 1/2)	Local delivery using gelatin sponges revealed no statistically significant differences in primary or assisted primary patency between the treatment and control groups	Conte et al. (2009) [60]
	Mesenchymal stem cell	Preclinical (Murine)	Direct injection into the adventitia significantly reduced the expression of *MCP-1* and HIF-1α, increased the mean luminal area, and reduced the mean neointimal area and neointimal cell density	Yang et al. (2015) [61]
		Clinical (Phase 1/2)	Ongoing trial	NCT02808208

Abbreviations: AVF, arteriovenous fistula; HD, hemodialysis; HIF-1α, hypoxia-inducible factor-1 alpha; IER3, immediate early response 3; MCP-1, monocyte chemoattractant protein-1; MP, microparticle; PLGA, polylactic-co-glycolic acid; TGF-β1, transforming growth factor-beta 1; VEGF-A, vascular endothelial growth factor-A.

## Data Availability

No new data were created or analyzed in this study. Data sharing is not applicable to this article.
